# Predicted declines in suitable habitat for greater one‐horned rhinoceros (*Rhinoceros unicornis*) under future climate and land use change scenarios

**DOI:** 10.1002/ece3.8421

**Published:** 2021-12-07

**Authors:** Ganesh Pant, Tek Maraseni, Armando Apan, Benjamin L. Allen

**Affiliations:** ^1^ Ministry of Forests and Environment Singhadurbar Kathmandu Nepal; ^2^ Institute for Life Sciences and the Environment University of Southern Queensland Toowoomba Qld Australia; ^3^ University of Sunshine Coast Sippy Downs Qld Australia; ^4^ Institute of Environmental Science and Meteorology University of the Philippines Diliman Quezon City Philippines; ^5^ Centre for African Conservation Ecology Nelson Mandela University Port Elizabeth South Africa

**Keywords:** BIOMOD2, climate change refugia, correlative approach, ensemble modeling, habitat loss, land use change, species distribution modeling

## Abstract

Rapidly changing climate is likely to modify the spatial distribution of both flora and fauna. Land use change continues to alter the availability and quality of habitat and further intensifies the effects of climate change on wildlife species. We used an ensemble modeling approach to predict changes in habitat suitability for an iconic wildlife species, greater one‐horned rhinoceros due to the combined effects of climate and land use changes. We compiled an extensive database on current rhinoceros distribution and selected nine ecologically meaningful environmental variables for developing ensemble models of habitat suitability using ten different species distribution modeling algorithms in the BIOMOD2 R package; and we did this under current climatic conditions and then projected them onto two possible climate change scenarios (SSP1‐2.6 and SSP5‐8.5) and two different time frames (2050 and 2070). Out of ten algorithms, random forest performed the best, and five environmental variables—distance from grasslands, mean temperature of driest quarter, distance from wetlands, annual precipitation, and slope, contributed the most in the model. The ensemble model estimated the current suitable habitat of rhinoceros to be 2610 km^2^, about 1.77% of the total area of Nepal. The future habitat suitability under the lowest and highest emission scenarios was estimated to be: (1) 2325 and 1904 km^2^ in 2050; and (2) 2287 and 1686 km^2^ in 2070, respectively. Our results suggest that over one‐third of the current rhinoceros habitat would become unsuitable within a period of 50 years, with the predicted declines being influenced to a greater degree by climatic changes than land use changes. We have recommended several measures to moderate these impacts, including relocation of the proposed Nijgad International Airport given that a considerable portion of potential rhinoceros habitat will be lost if the airport is constructed on the currently proposed site.

## Graphical abstract

We developed ensemble models to predict the changes in suitable habitats for rhinoceros in Nepal. We estimated that about 1.77% of the total area of Nepal is currently suitable for rhinoceros. Our results indicate that over one‐third of the current rhinoceros habitat would become unsuitable within a period of 50 years.

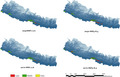

## 1 INTRODUCTION

Climate plays an important role in determining the distribution of species over space and time, and the species thrive only in a particular environment because they are adapted to a certain climatic condition in their geographical range (Araújo & Pearson, 2005; Choudhury et al., 2016). The earth's temperature has increased by about 0.74°C in the last 100 years, and the global average temperature is projected to rise further by 4.3 ± 0.7°C by 2100 (Almazroui et al., 2020; IPCC, 2014). Such climate warming is anticipated to have many far‐reaching consequences for global biodiversity and associated ecosystem functions (Hannah et al., 2005; IPBES, 2019; Pacifici et al., 2017) including (1) increased rates of species extinction (Fulton, 2017; Pearson et al., 2014; Thomas et al., 2004), (2) population decline (Both et al., 2006; Moritz & Agudo, 2013; Soroye et al., 2020), (3) changes in phenology (Cohen et al., 2018; Menzel et al., 2020; Zhixia et al., 2020), (4) increased invasion by alien species (Gong et al., 2020; Hulme, 2017; Wallingford et al., 2020), and (5) range shifts and decline in habitat suitability of species (Corlett, 2015; Thuiller et al., 2011; Trisos et al., 2020). More specifically, climate change may push some species to higher elevations and the species adapted to live on mountains are particularly vulnerable to the likely impacts of climate change (Aryal et al., 2016; Chen et al., 2011; Elsen et al., 2020). It is predicted that loss of habitat, changes in species distribution, and increased extinction of species will continue if we fail to address the likely consequences of the changing climate (Hannah et al., 2020), while climate‐induced habitat alteration will further endanger global biodiversity (Bellard et al., 2012; Erdelen, 2020; Pires et al., 2018). On the other hand, habitat loss and fragmentation due to land use changes are likely to exacerbate the effects of climate change on species and ecological dynamics across the globe (Kaszta et al., 2020; Oliver et al., 2015).

**FIGURE 1:**
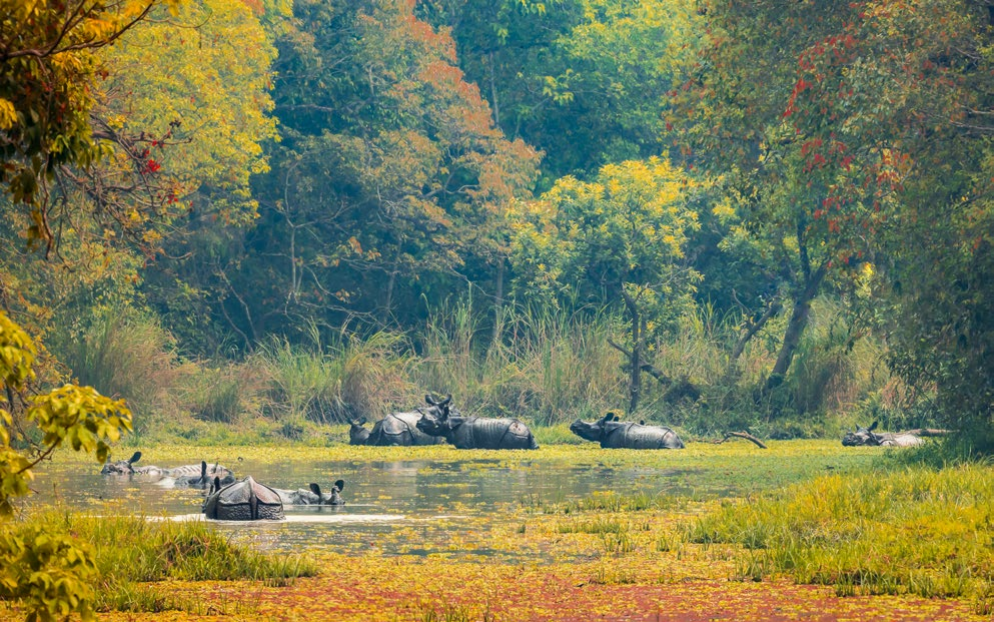
Greater one‐horned rhinoceros (*Rhinoceros unicornis*) in Chitwan National Park, Nepal (Photo credit: Sagar Giri)

Greater one‐horned rhinoceros (*Rhinoceros unicornis*, hereafter “rhinoceros”) is a threatened megaherbivore, currently surviving in a few protected areas in the northern foothills of India and the southern parts of Nepal (Ellis & Talukdar, 2019; Pant et al., 2020b). In Nepal, Chitwan National Park is a prime habitat for rhinoceros (Figure 1) and a small population of which was translocated to Bardia and Shuklaphanta National Parks from Chitwan (DNPWC, 2017). Rhinoceroses were abundant until the nineteenth century (Foose & Strien, 1997), before the population in the wild sharply declined to approximately 500 individuals during the early 1960s (Rookmaaker et al., 2016). Following intensive conservation efforts since then the rhinoceros population in both India and Nepal has been gradually recovering, and there are approximately 3550 rhinoceros today (Ellis & Talukdar, 2019). Rhinoceroses are habitat specialists and prefer a mosaic of grassland patches dominated by *Saccharum spontaneum* and the riverine forests on alluvial floodplains along the foothills of the Himalayas, where green growth and water remain available all year round (Dinerstein & Price, 1991; Jnawali, 1995; Laurie, 1982; Pradhan et al., 2008). The inadequacy of currently available habitat is identified as a challenge for rhinoceros conservation (Pant et al., 2020b), and the decrease in both quality and quantity of rhinoceros habitat has been observed in protected areas in both countries, which is likely to deteriorate in future and is thus likely to affect its survival (Medhi & Saha, 2014; Sarma et al., 2009; Subedi, 2012). Despite its population recovery, rhinoceros is facing conservation challenges due to habitat loss in terms of fragmentation and encroachment and the problem is likely to be intensified in future due to the impacts of climate change (DNPWC, 2017; Pant et al., 2020b). Although a few researchers have recently begun studying rhinoceros in relation to climate change (Adhikari & Shah, 2020; Mukherjee et al., 2020; Pant et al., 2020a), the likely consequences of the changing climate on rhinoceroses and their habitat are not well understood (DNPWC, 2009; Pant et al., 2020b).

Species distribution modeling (SDM), which is also known as ecological niche modeling, establishes a species–environment relationship to explain and predict the probable distribution of a species (Elith & Leathwick, 2009; Thuiller et al., 2009). It can be used as a correlative approach of assessing vulnerability of a species to climate change, which provides spatial information regarding the potential climate change impacts on species (Foden & Young, 2016). The SDM has the potential to achieve conservation planning goals by helping to widen our knowledge of species distribution (Franklin, 2010; Jetz et al., 2012; Raymond et al., 2020) and predicting the impacts of climate change on species (Araújo et al., 2005; Berry et al., 2002; Elith et al., 2010). Likewise, SDM helps in projecting species distribution in space and time, which is central to extinction risk analysis (Elith & Leathwick, 2009). SDMs for predicting future events are an especially useful tool for prioritizing biodiversity conservation (Araújo et al., 2005; Bellard et al., 2012). However, the predictive performance of modeling techniques differs, and the uncertainty of predictions could be substantially reduced by using consensus methods (Marmion et al., 2009). These ensemble techniques of SDM systematically evaluate the species distribution models and its potential variations under future climate change, and BIOMOD serves as a suitable platform to such modeling (Thuiller et al., 2009). Using an ensemble approach, SDM can combine predictions from many modeling techniques and the predictive performance is believed to be improved considerably (Hao et al., 2020).

Here, we explored the likely vulnerability of rhinoceros in Nepal due to the combined effects of climate and land use changes using ensemble SDM techniques. Our specific objectives included (1) identifying the ecological niche of rhinoceros in Nepal, (2) investigating the impacts of different climate and land use change scenarios on future habitat suitability of rhinoceros, and (3) identifying the climate change refugia to secure the future persistence of rhinoceros in a changing climate. Previous studies on rhinoceros habitat suitability (Kafley et al., 2009; Rimal et al., 2018; Thapa et al., 2014) identified only current habitat at selected sites, while Adhikari and Shah (2020) has also predicted future suitable habitat throughout Nepal using bioclimatic and topographic data as predictor variables. In contrast, our study identified current suitable habitat for rhinoceros and predicted future habitat for all of Nepal under two different climate and land use change scenarios using bioclimatic, topographic, habitat, and anthropogenic data as predictor variables.

## 2 METHODS

### 2.1 Study area

**FIGURE 2:**
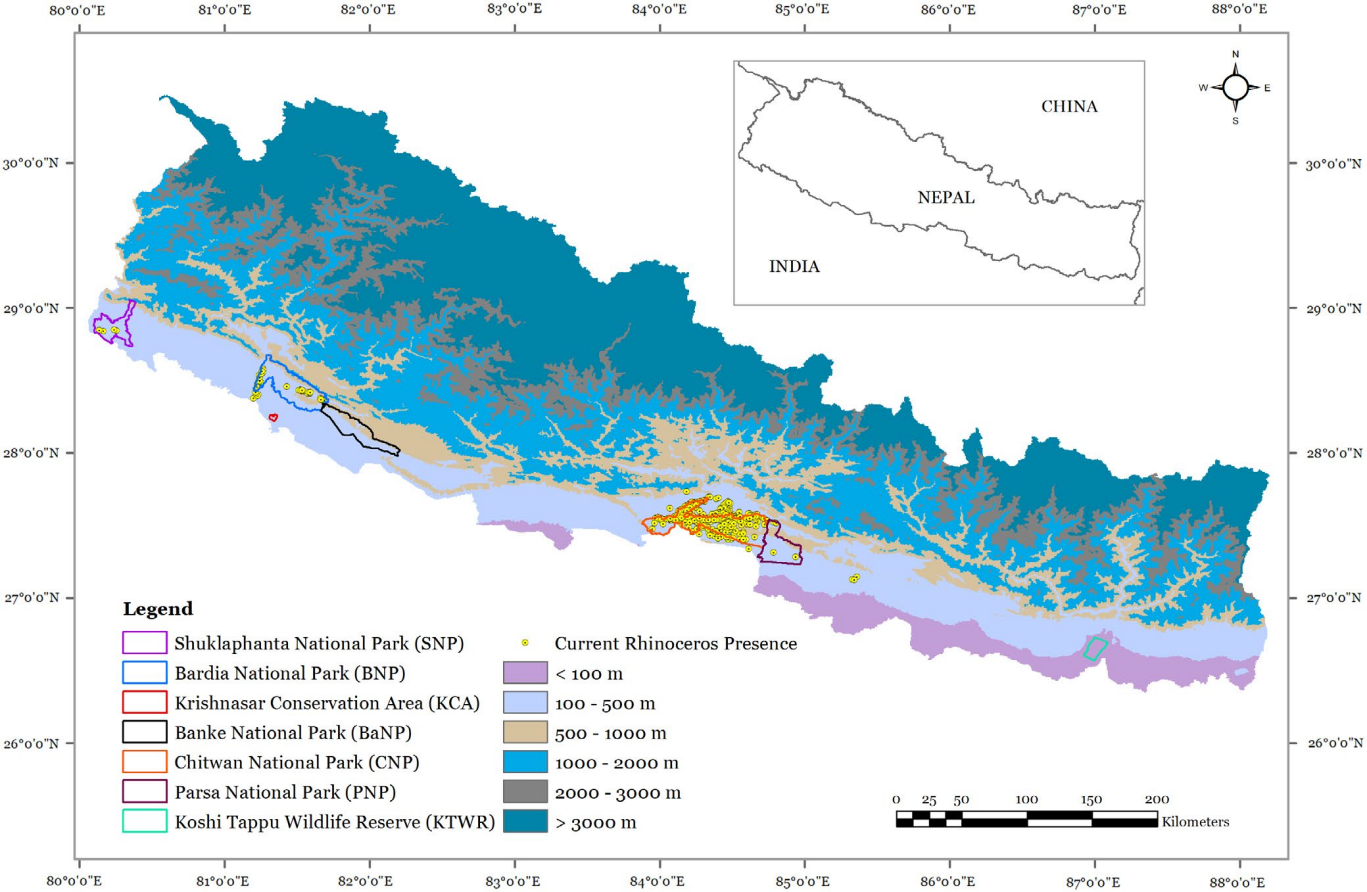
Study area map showing the current distribution of greater one‐horned rhinoceros and elevation range in Nepal

Nepal extends over 147,516 km^2^ in South Asia between latitudes of 26°22′ to 30°27′ north and longitudes of 80°04′ to 88°12′ east. It is endowed with rich biodiversity because of its varied climate and topography along a sharp altitudinal gradient ranging from 60 to 8848 m above mean sea level (Figure 2) within a north–south span of about 140 km (Bhattacharjee et al., 2017; Paudel et al., 2012). Nepal is divided into three major physiographical regions: (1) lowland (Terai and Siwalik) (2) mid‐hills, and (3) high mountain (Shrestha & Aryal, 2011). The climate is dominated by the south‐easterly monsoon, and most of the precipitation occurs during the rainy summer months between June and September (Shrestha & Aryal, 2011; Shrestha et al., 2000). The annual mean temperature is 18°C and the average annual precipitation is 1768 mm (Shrestha et al., 2000). Rhinoceroses in Nepal are confined to alluvial flood plains in the southern lowlands (DNPWC, 2017). There are seven protected areas (PAs) in the lowlands of Nepal namely Shuklaphanta National Park (SNP), Bardia National Park (BNP), Banke National Park (BaNP), Krishnasar Conservation Area (KCA), Chitwan National Park (CNP), Parsa National Park (PNP), and Koshi Tappu Wildlife Reserve (KTWR). Of these seven PAs, SNP, BNP, CNP, and PNP have rhinoceros at present.

### 2.2 Rhinoceros presence data

Records of rhinoceros presence modeled in our study were obtained mostly from national census and periodic monitoring data held by the Department of National Parks and Wildlife Conservation (DNPWC), Nepal, between 2008 and 2017 (Table 1). We also collected a small number of additional opportunistic rhinoceros presence records from fieldwork conducted specifically for this research in April 2019, as well as from an online database, the Global Biodiversity Information Facility (GBIF). In summary, we compiled an extensive database of 2739 current rhinoceros presence points. In the next step, we cleaned the presence data removing the duplicates and the points appeared outside the known distribution range of the species.

**TABLE 1:** Records of species presence compiled from various sources and used for species distribution modeling for greater one‐horned rhinoceros in Nepal

Data	Year	Presence points	Source
National rhinoceros census	2008	423	Department of National Parks and Wildlife Conservation
	2011	503	
	2015	645	
Rhinoceros monitoring in Babai Valley, Bardia	2016	183	Bardia National Park
GPS points from collared rhinoceros in Chitwan	2017	844	Chitwan National Park
Fieldwork for this study	2019	56	Self
GBIF Database	2020	85	GBIF website
Total		2739	

Abbreviations: GBIF, Global Biodiversity Information Facility; GPS, Global Positioning System.

We used the SpThin package in R to spatially rarefy the occurrence dataset to ensure that no two points were within a grid of 1 × 1 km (Aiello‐Lammens et al., 2015), given that the spatial resolution of the environmental variables used in this modeling was 1 km. Hence, we retained only one presence point in each grid cell to reduce spatial autocorrelation and avoid the inflated measures of accuracy (Veloz, 2009). Spatial filtering also reduces the effects of sample bias and helps to improve the predictive performance of the models (Boria et al., 2014). After filtering, a set of 495 spatially independent locations of rhinoceros presence were retained and used for modeling. We did not use historical presence records of rhinoceros given that most of the environmental variables we used have substantially changed when compared to historical periods. Besides, our focus was to identify current and future suitable habitat that are available for rhinoceros conservation, not the historical range of the species. Historical period in the case of rhinoceros in Nepal is before 1970s as its habitat was almost entirely lost to agriculture during the early 1960s and occurring only in a few isolated protected areas from the 1970s onward (DNPWC, 2017; Subedi et al., 2017).

### 2.3 Environmental variables

We used a combination of bioclimatic, topographic, habitat, and anthropogenic variables to predict current and future suitable habitat for rhinoceros in Nepal. We endeavored to include meaningful predictor variables given that variable selection is considered a vital step in SDM (Araujo & Guisan, 2006). First, we identified a set of 28 variables (Appendix S1) primarily based on literature suggesting the significance of these variables for rhinoceros habitat suitability (Dinerstein, 2003; Dinerstein & Price, 1991; Jnawali, 1995; Laurie, 1982; Pant et al., 2020b; Pradhan et al., 2008; Subedi, 2012). We then excluded those environmental variables with correlation coefficients >0.8 and variance inflation factor (VIF) >5 after testing the multicollinearity among environmental variables using the USDM (Uncertainty Analysis for Species Distribution Models) package in R to avoid model overfitting (Gareth et al., 2013; Naimi et al., 2014), retaining 14 variables for further analysis (Appendix S2). Finally, we selected nine of these as ecologically meaningful variables and used them as predictor variables in habitat suitability modeling for rhinoceros (Table 2) following a reiterative process of model formation and stepwise removal of the least contributing variables, as suggested by Zeng et al. (2016). The main purpose of reducing the number of environmental variables is to enhance the predictive performance of the model given that ensemble models avoid overfitting without losing explanatory power through reducing the number of predictor variables (Breiner et al., 2015). We projected all variables to WGS84 and resampled these raster data in ArcMap 10.8.1 (ESRI, 2020) using bilinear interpolation method at a spatial resolution of 1 km, given that data from various sources were in different grain size ranging from ~10 m to ~1 km resolution.

**TABLE 2:** Environmental variables used for habitat suitability modeling for greater one‐horned rhinoceros in Nepal

Category	Source	Selected variables	Resolution	Type
Bioclimatic	WORLDCLIM	BIO7—Temperature annual range	~1 km	Continuous
		BIO9—Mean temperature of driest quarter	~1 km	Continuous
		BIO12—Annual precipitation	~1 km	Continuous
Topographic and habitat	SRTM	Slope	~ 30 m	Continuous
	ESRI 2020 Land Cover	Distance from grasslands	~10 m	Continuous
		Distance from wetlands	~10 m	Continuous
		Distance from forests	~10 m	Continuous
Anthropogenic	MODIS Land Cover	Croplands	~500 m	Continuous
	HDX	Population density	~1 km	Continuous

Abbreviations: HDX, Humanitarian Data Exchange; MODIS, Moderate Resolution Imaging Spectroradiometer;SRTM, Shuttle Radar Topographic Mission.

#### 2.3.1 Bioclimatic variables

Bioclimatic variables are widely used for spatial modeling given that these are ecologically meaningful and describe annual trends, seasonality, and extremes of temperature and precipitation (Hijmans et al., 2005; Hijmans, 2012). Rhinoceroses prefer moist habitats with moderate climate (Subedi, 2012), and their occurrence was recorded from areas having >1500 mm average annual rainfall and >22°C annual mean temperature (Dinerstein & Price, 1991; DNPWC, 2017; Laurie, 1982). We downloaded 19 bioclimatic variables for the current climate (1970–2000) from WorldClim— Global Climate Data (Fick & Hijmans, 2017). Rhinoceros shows affinity toward higher rainfall and moderate temperature (Pant et al., 2020b; Subedi, 2012).

#### 2.3.2 Topographic and habitat variables

The current distribution of rhinoceros is recorded from 100 to 500 m elevation in and around four protected areas located in the southern part of Nepal (DNPWC, 2009; Pant et al., 2020b). It is evident from other studies that the topographic variables, such as elevation, and slope have an influence on habitat suitability of megaherbivores (Sarma et al., 2020). Thus, we included topographic data as one of the predictor variables in our models. We derived elevation data from Shuttle Rader Topographic Mission (SRTM) Digital Elevation Model (DEM) of 30 m spatial resolution downloaded from the United States Geological Survey database (USGS, 2020) from which aspect and slope data were computed using ArcMap 10.8.1 (ESRI, 2020).

Rhinoceros, primarily a grazer, is a grassland dependent species, it prefers riverine forests, and it further requires waterholes to wallowing for thermoregulation (Dinerstein & Price, 1991; Laurie, 1978). Thus, grasslands, riverine forests, and wetlands play a fundamental role in determining the habitat suitability of this species. Therefore, we extracted the layers containing grasslands, forests, and wetlands of the study area from Esri 2020 Land Cover (Karra & Kontgis, 2021). We generated raster data layers containing proximity to grasslands, forests, and wetlands using Euclidean Distance tool in ArcMap 10.8.1 (ESRI, 2020).

#### 2.3.3 Anthropogenic variables

Anthropogenic activities influence the species distribution and have been identified as a threat to rhinoceros (DNPWC, 2017; Pant et al., 2020b), and these were also incorporated into our model. Anthropogenic variables used were croplands and human population density. To include the land use change scenarios, we extracted the combined class of croplands and cropland/natural vegetation mosaics from Moderate Resolution Imaging Spectroradiometer (MODIS) Land Cover Type (MCD12Q1) Version 6 (Friedl & Sulla‐Menashe, 2019). Likewise, human population density data were downloaded from the Humanitarian Data Exchange Dataset (HDX, 2020).

#### 2.3.4 Future climate and land use change scenarios

We used the future bioclimatic variables from Models for Interdisciplinary Research on Climate (MIROC), particularly MIROC6, to model the response of rhinoceros to future climate. MIROC6 is the recently updated version of MIROC5 (Michibata et al., 2019), and the overall reproducibility of mean climate and internal variability in MIROC6 is better than that in its previous version (Tatebe et al., 2019). The MIROC5 is a consistent global circulation model (GCM) for rainfall projection in the Indian subcontinent (Babar et al., 2015) which simulates extreme and summer precipitation better than other GCMs for the South Asian region (Mishra et al., 2014). MIROC5 is also capable of capturing the distribution and variability of temperature in this region (Yu et al., 2015). Thus, MIROC6 was selected for this study considering the better performance of this model in predicting future climate over the geographical range of rhinoceros. Data are available for four Shared Socioeconomic Pathways (SSPs), where SSP1‐2.6 is based on a lower emission scenario, which anticipates a mean warming of well below 2°C by 2100, while SSP5‐8.5 is based on the highest emission scenario, with a mean warming of 5.5°C by the end of this century (Hausfather, 2018). In this study, we have chosen SSP1‐2.6 and SSP5‐8.5 to model the suitable habitat for rhinoceros to capture the full range of predicted climate change scenarios.

We used data on global land use and land cover change simulation for years 2050 and 2100 from the GeoSOS global database to project the future scenarios for human land use changes (Li et al., 2017). This simulation has combined MODIS land cover categories into six classes and predicted the changes from 2010 to 2100 under four scenarios of the Intergovernmental Panel on Climate Change (IPCC, 2014) Special Report on Emission Scenarios using Future Land Use Simulation (FLUS) system. We extracted the land use category “farmland” of Li et al. (2017) which has combined two categories: (i) croplands and (ii) cropland/natural vegetation mosaics from MODIS land cover. We included two land use change scenarios: A1B (moderate increase in land use across all resources) and A2 (high emphasis on development with adverse impact on the environment). We grouped SSP1‐2.6 with A1B scenario and SSP5‐8.5 with A2 scenario while predicting the rhinoceros habitat suitability for 2050 and 2070 due to the combined effects of climate and land use changes.

### 2.4 Species distribution modeling methodology

**FIGURE 3:**
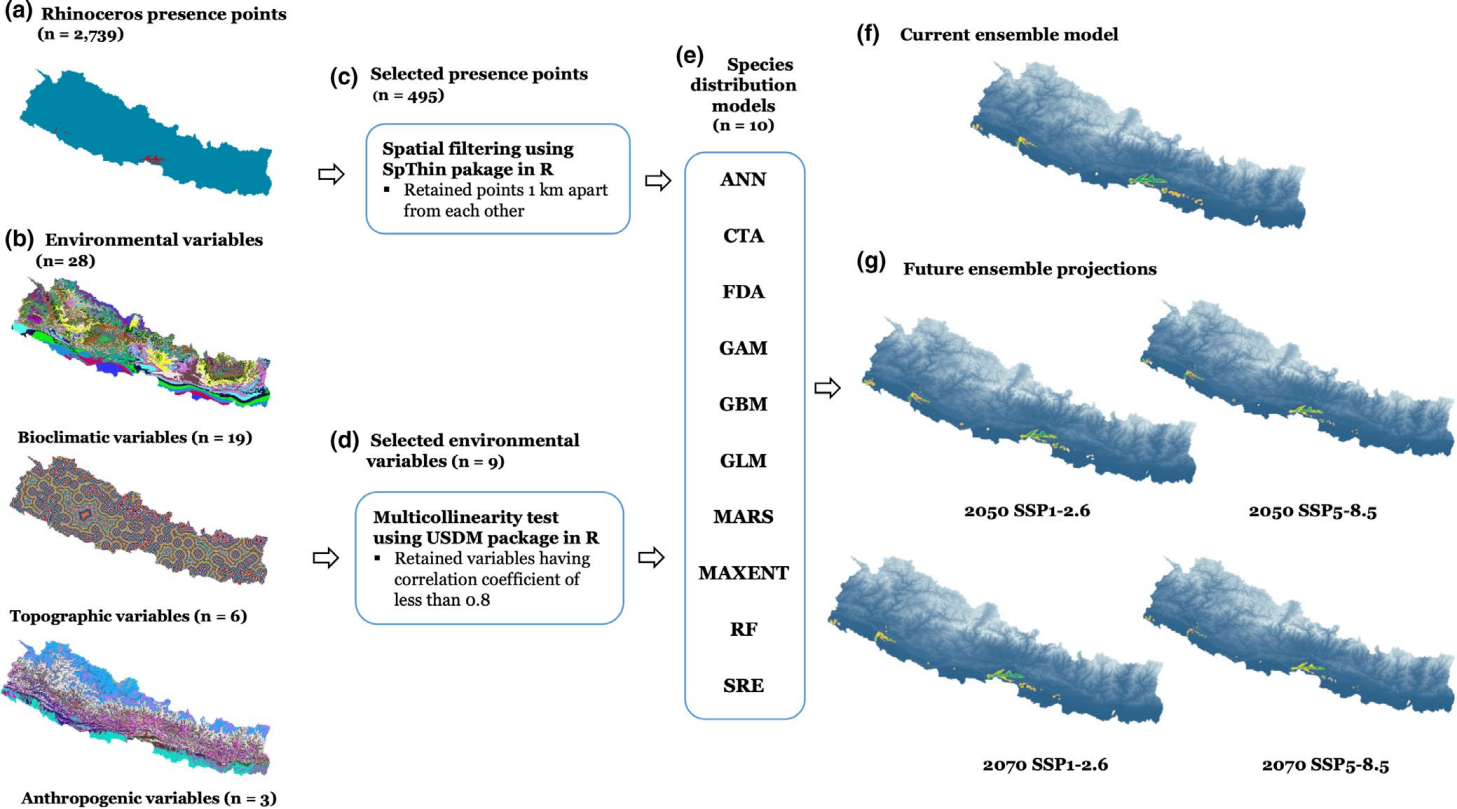
Methods used for ensemble species distribution modeling for greater one‐horned rhinoceros in Nepal using BIOMOD2 package in R (a–e); current ensemble model (f), ensemble projections into future greenhouse gas (GHG) emission scenarios (g). SSP1‐2.6 and SSP5‐8.5 are two different climate change scenarios that anticipate a mean warming of 2 and 5.5°C by 2100, respectively

We followed the overview, data, model, assessment, and prediction (ODMAP) protocol proposed by Zurell et al. (2020) in developing habitat suitability models for rhinoceros in Nepal (Appendix S3). Combining several models generated from different modeling techniques into an ensemble map is highly acknowledged in recent SDM exercises given its better predictive accuracy (Hao et al., 2019). Thus, we used an ensemble modeling approach to develop habitat suitability models for rhinoceros in Nepal. We generated ensemble models based on ten algorithms: artificial neural network (ANN), classification tree analysis (CTA), flexible discriminant analysis (FDA), generalized additive model (GAM), generalized boosting model (GBM), generalized linear model (GLM), multiple adaptive regression splines (MARS), maximum entropy (MAXENT), random forest (RF), and surface range envelope (SRE) using the BIOMOD2 package (Thuiller et al., 2020) in R (R Development Core Team, 2020), as shown in Figure 3. First, data layers were prepared in ArcMap 10.8.1 (ESRI, 2020) and the multicollinearity among bioclimatic variables was tested. After selecting the appropriate data layers, the models were calibrated to generate suitability maps. Rhinoceros presence and pseudo‐absence data were split into training (80%) and testing data sets (20%). With the training dataset, we randomly generated 10,000 pseudo‐absence points as suggested by Barbet‐Massin et al. (2012), in which we assigned equal weight for the presence and pseudo‐absence datasets, and we repeated the pseudo‐absence generation three times to avoid random bias. This modeling, comprising ten algorithms, three pseudo‐absence selection, and three evaluation runs resulted into a total of 90 model runs. We generated ensemble models using the ensemble modeling function in BIOMOD2. Finally, we employed range size function within the BIOMOD2 package when calculating the range shifts for rhinoceros under different climate and land use change scenarios in Nepal.

### 2.5 Model evaluation and validation

Model evaluation and validation in SDM examine the accuracy of the model prediction. It assesses the predictive performance of a model based on various evaluation statistics and is generally performed using response curves, variable importance, and model coefficients. The area under the receiver operating characteristics (ROC) curve known as area under the curve (AUC) is a standard method to assess the accuracy of predictive distribution models (Lobo et al., 2008). Likewise, true skill statistics (TSS) is a common method to evaluate the predictive performance of such models (Allouche et al., 2006). These two methods are independent, but it is desirable to execute both methods for cross checking (Thuiller et al., 2009). We therefore used TSS to evaluate the predictive performance while we analyzed AUC for cross‐comparison of our models. The TSS value accounts for both omission as well as commission errors, which ranges from +1 to −1 (Allouche et al., 2006). The model is considered perfect if the TSS value is +1, whereas the TSS value between 0.7 and 0.9 indicates a good model (Allouche et al., 2006; Thuiller et al., 2009). In addition, we employed cross validation techniques such as the Boyce index to further assess the predictive performance of the models (Boyce et al., 2002; Engler et al., 2004), which is the most appropriate evaluation metric in the case of presence‐only models (Hirzel et al., 2006). We selected all models having a TSS value >0.85 for building ensemble model using the weighted mean approach. Consensus method based on weighted mean approach increases the model accuracy (Marmion et al., 2009). The weighted mean approach creates the final model based on the selected threshold of the TSS value and generates the binary map which is also known as the presence–absence map.

We classified the output map into three suitability classes: low (<60%), moderate (60–80%), and high (>80%) using the reclassify function in ArcMap 10.8.1 (ESRI, 2020). In addition, we further validated the on‐ground reality of the current habitat suitability model for rhinoceros in Nepal through expert consultation. For this, we shared the current habitat suitability model we generated to five field biologists each having more than 10 years of professional experience in research and management of rhinoceros in Nepal. All of them agreed that the current suitability model has captured not only the areas currently occupied by rhinoceros but also the potential habitat having similar environmental conditions at present that are likely to support rhinoceros populations in Nepal.

## 3 RESULTS

### 3.1 Model performance and contribution of predictor variables

**FIGURE 4:**
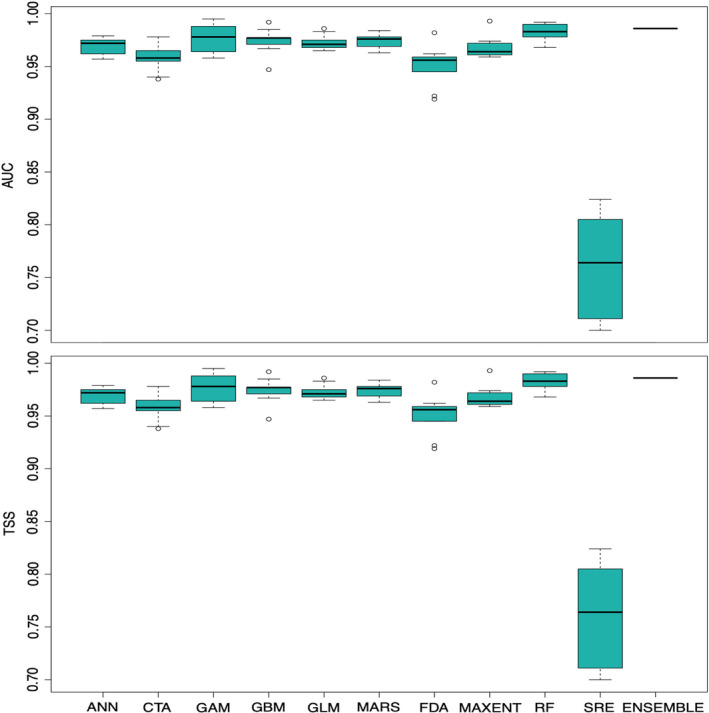
Predictive performance of different modeling techniques used for species distribution modeling of greater one‐horned rhinoceros in Nepal, based on area under curve (AUC) and true skill statistics (TSS) value. The AUC and TSS values of the ensemble model are also shown for comparison

**FIGURE 5:**
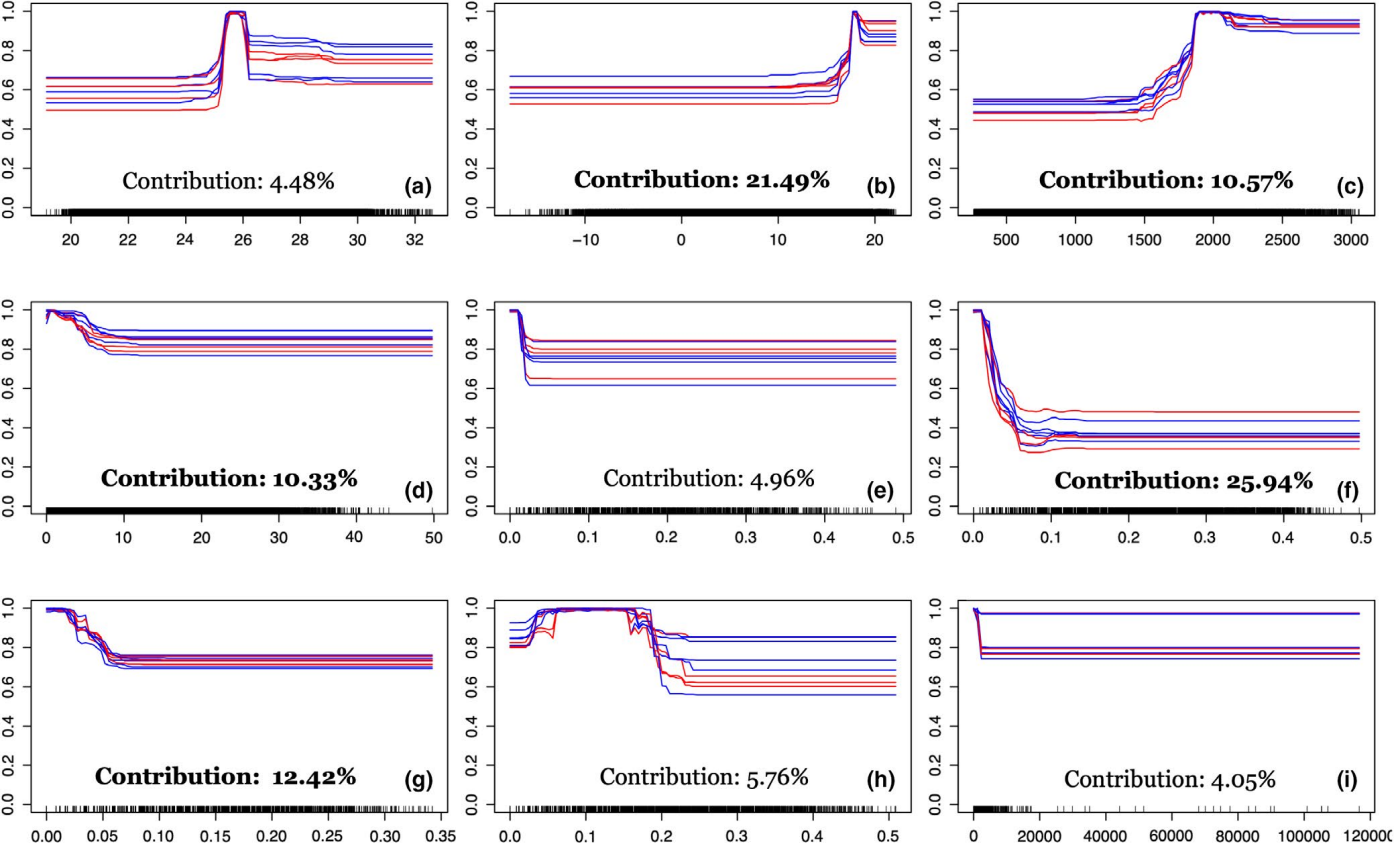
Response curve of environmental variables used to model habitat suitability of greater one‐horned rhinoceros in Nepal (a) temperature annual range (BIO7), (b) mean temperature of driest quarter (BIO9), (c) annual precipitation (BIO12), (d) slope, (e) distance from forests, (f) distance from grasslands, (g) distance from wetlands, (h) croplands, and (i) population density

The predictive performance of our ensemble model was excellent, with a TSS value of 0.986. Likewise, all the ten algorithms had an average TSS value of >0.750. SRE had the lowest TSS value (0.763), while RF had the highest TSS value (0.983) (Figure 4). Similarly, AUC value of the ensemble model was 0.999 whereas RF had the highest (0.998) and SRE had the lowest (0.882) AUC value. Environmental variables contributed differently to our models (Figure 5), but the variables that contributed the most were distance from grasslands, mean temperature of driest quarter (BIO9), distance from wetlands, annual precipitation (BIO12), and slope. As expected, distance from grasslands had the highest contribution (25.94%) to our model, followed by the mean temperature of driest quarter (21.49%) (Figure 5b,f). The distance from wetlands contributed 12.42% in our model and the habitat suitability decreased with increasing distance from wetlands (Figure 5g). Response curves showed that areas with >1500 mm of annual rainfall were suitable for rhinoceros and this covariate contributed 10.57% in the model (Figure 5c). The fifth most contributing variable was slope (10.33%), indicating that slopes of <10° were most suitable for rhinoceros (Figure 5e). The remaining four variables collectively contributed 19.25% in the model (Figure 4a,e,h,i).

### 3.2 Rhinoceros habitat suitability

**FIGURE 6:**
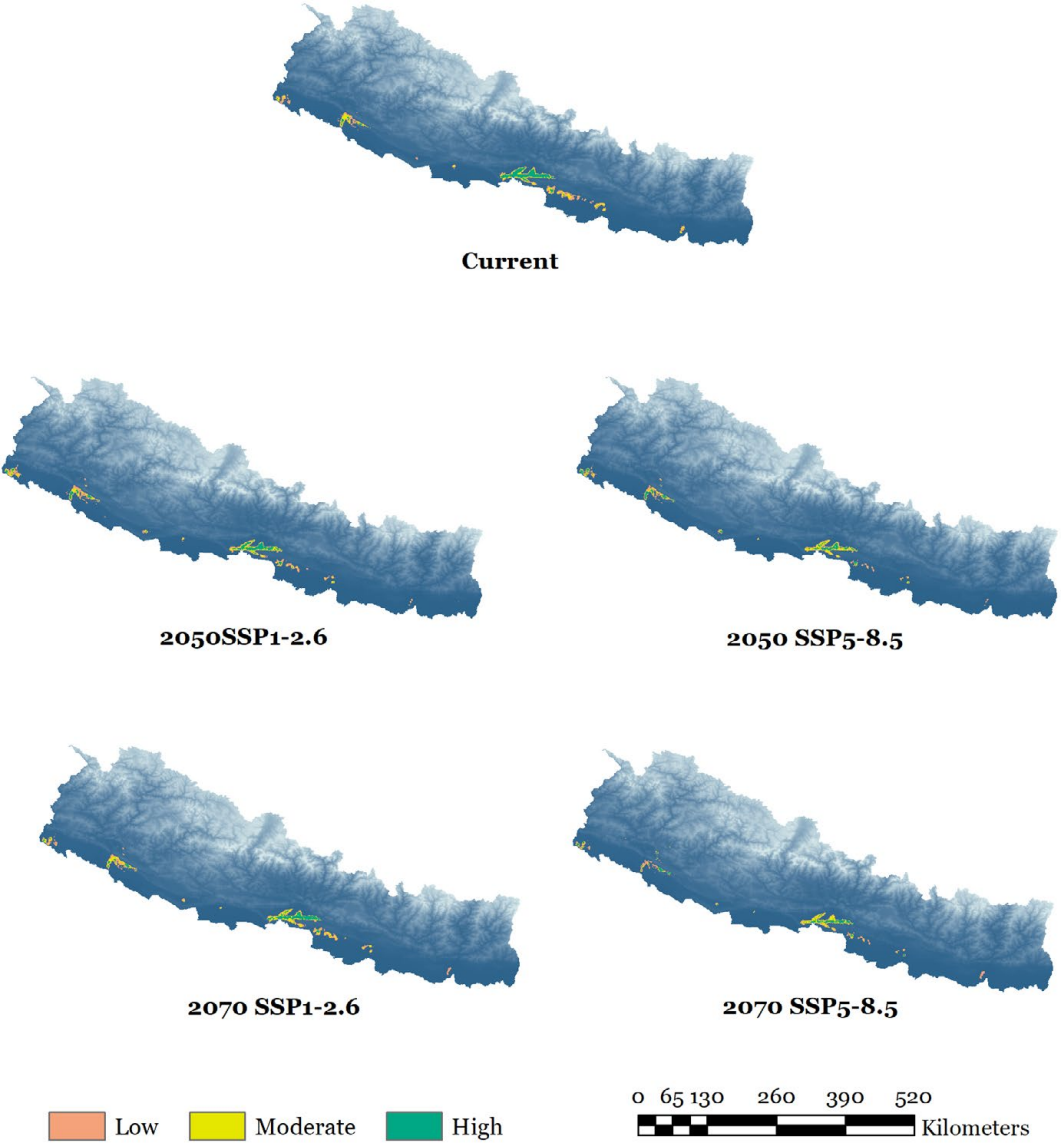
Extent of suitable habitat for greater one‐horned rhinoceros in Nepal under current and future climate and land use change scenarios

The extent of habitat suitability for rhinoceros in Nepal under current and future climate change scenarios is presented in Figure 6. The estimated current suitable habitat for rhinoceros is 2610 km^2^, which is 1.77% of the total area of Nepal. Of current suitable habitat, 2044 km^2^ (78%) is inside protected areas (PAs) while the remaining 566 km^2^ (22%) lies outside PAs (Appendix S7 and S8 **)**. Among the five PAs and their buffer zones that are suitable for rhinoceros, CNP and KTWR have the highest (1063 km^2^) and the lowest suitable area (67 km^2^), respectively. The current suitable habitat of rhinoceros in BNP, PNP, and SNP is 447 km^2^, 291 km^2^, and 176 km^2^, respectively. At present, the model does not reveal any suitable rhinoceros habitat in KCA and BaNP. Most of the current suitable habitat of rhinoceros outside protected areas extends over Bara, Rautahat, Sarlahi, and Kapilbastu districts, although suitable rhinoceros habitat is distributed across 16 districts of Nepal. Of these 16 districts, Chitwan has the highest (904 km^2^) whereas Kailali, Surkhet, and Jhapa have negligible current suitable habitat (Appendix S9 and S10).

**FIGURE 7:**
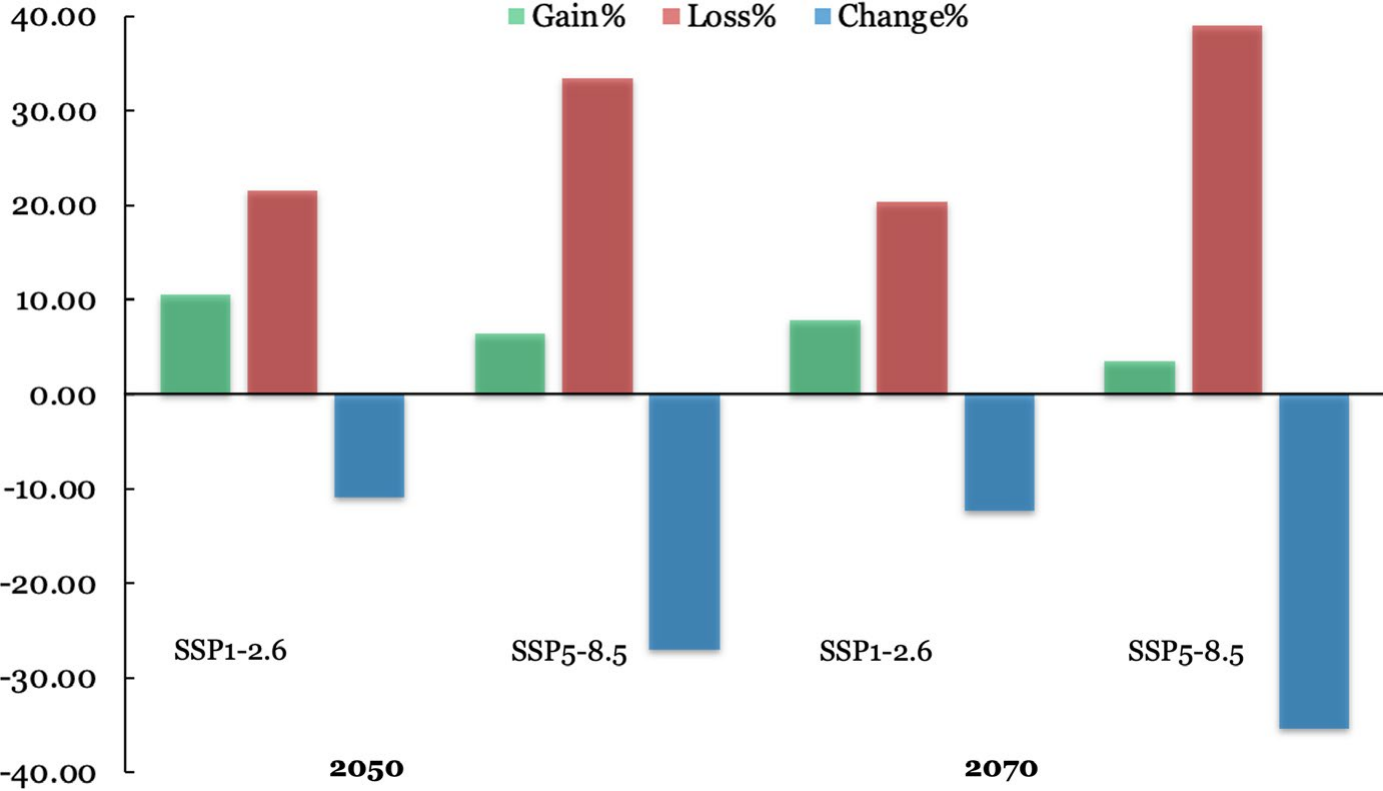
Percentage change in suitable habitat of greater one‐horned rhinoceros in Nepal predicted by the ensemble model under future climate and land use change scenarios

**FIGURE 8:**
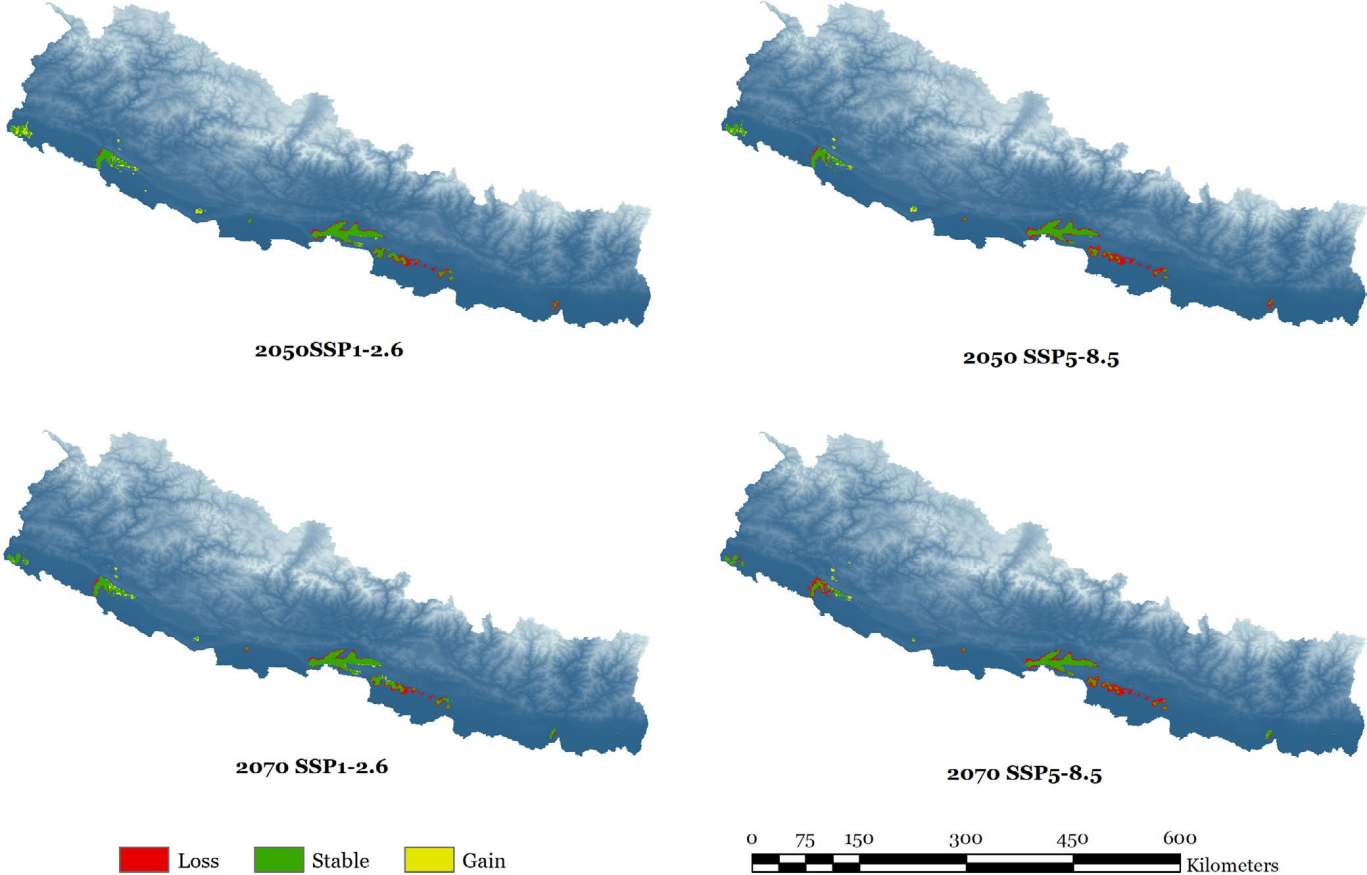
Extent of the predicted changes in suitable habitat for greater one‐horned rhinoceros in Nepal

A summary of suitable habitat areas for rhinoceros in Nepal under current and future climate and land use change scenarios estimated by the ensemble models is presented in Table 3. Under the SSP1‐2.6 scenario for 2050, a net loss of 285 km^2^ in suitable habitat is likely to occur and the highest reduction in suitable habitat (924 km^2^) is predicted under the SSP5‐8.5 scenario for 2070. The predicted change in habitat suitability of rhinoceros in Nepal under different climate and land use change scenarios by the end of 2070 is presented in Figures 7 and 8. In 2070, we predicted a net loss of 12.39% (323 km^2^) in current suitable habitat under SSP1‐2.6 climate scenario based on the predicted loss of 20.30% (539 km^2^) and a gain of 7.91% (206 km^2^) Likewise, 27.04% (706 km^2^) of the current suitable habitat of rhinoceros will be lost owing to a predicted loss of 33.42% (872 km^2^) and a gain of 6.39% (167 km^2^) under SSP5‐8.5 climate scenario in 2050.

**TABLE 3:** Estimated area of suitable habitat for greater one‐horned rhinoceros in Nepal under current and future climate and land use change scenarios

Climate scenario	Suitable habitat area (km^2^)				Percentage (%) of Nepal's area
	Low	Moderate	High	Total	
Current	1129	726	755	2610	1.77
2050 SSP1‐2.6	1082	651	592	2325	1.58
2050 SSP5‐8.5	832	616	456	1904	1.29
2070 SSP1‐2.6	1007	741	539	2287	1.55
2070 SSP5‐8.5	781	550	355	1686	1.14

Abbreviation: SSP, Shared Socioeconomic Pathways.

## 4 DISCUSSION

### 4.1 Model performance and contribution of predictor variables

The AUC and TSS values of the ensemble model were 0.986 and 0.999, respectively, indicating that our model was statistically robust, and the predictive performance was near perfect (Allouche et al., 2006). We endeavored to minimize the effects of uncertainties by spatially rarefying the presence points, use of minimum number of environmental variables and applying cross‐validation techniques (Breiner et al., 2015; Hijmans, 2012). For instance, we used 80% of the presence and pseudo‐absence datasets for model calibration and the remaining 20% of data was used for model evaluation, generated evaluation metrics from independently divided testing and evaluating datasets, and used the Boyce index for cross‐validation. Lobo et al. (2008) suggested that AUC value of over 0.8 is likely to be an indication of overparameterization. However, the AUC and TSS values from testing and evaluating data indicated the consistent predictive performance of our models (Appendix S4 and S5). Likewise, we compared the AUC values of our models to the Boyce index (Appendix S6) which also showed that all these models are performing well. For example, the RF model which performed the best in our data had the AUC and the Boyce index of 0.998 and 0.994, respectively. The suitability map generated has captured the current habitat of rhinoceros well and all the models are consistently performing in different presence–absence data and various model runs. Hence, we believe that our model has not been affected from overfitting.

Our ensemble approach identified suitable rhinoceros habitat that was mainly concentrated in the central and western lowland of Nepal, indicating that its distribution was constrained by topographic variables. Suitable habitat ranges of many terrestrial species have shifted toward higher elevations in response to changing climate (Chen et al., 2011; Dar et al., 2021; Moritz et al., 2008). Rhinoceros habitat suitability is limited by topographic factors given that slope contributed strongly to our models (Figure 4e). We excluded the elevation data in our model due to its high correlation with other variables, but instead used slope as a proxy for elevation in interpreting the results given that slope increases with increasing elevation in Nepal. Currently, the known distribution of rhinoceros in Nepal extends between the elevation range of 100 and 500 m (DNPWC, 2009; Pant et al., 2020b), consistent with our findings. Rhinoceroses are not likely to shift into higher elevations like some other species but instead appear trapped in small patches of suitable habitat at lower elevations.

The distance from grasslands, mean temperature of driest quarter, distance from wetlands, annual precipitation, and slope were the predictor variables with the strongest influence in our model, whereas human population density and changes in croplands as an anthropogenic variable had only a slight contribution (Figure 5h,i). Even though temperature and precipitation patterns are strong determinants of rhinoceros habitat suitability, the coarse spatial resolution of these covariates may obscure the interplay between these climatic factors and the actual suitability of the habitat for rhinoceros. Given that a finer resolution is likely to increase model accuracy (Connor et al., 2018), the inclusion of site‐specific climate characteristics, terrain attributes, and anthropogenic data at finer grain sizes for model building possibly results in better accuracy in prediction of rhinoceros habitat suitability. Regardless, any such refinements to our model are unlikely to produce wholesale differences to the gross species distribution predictions we have made, and rhinoceros will still be trapped in small habitat patches in lower elevations.

### 4.2 Rhinoceros habitat suitability

Our results show that 35% of the current suitable habitat will be lost by 2070 due to the combined effects of climate and land use changes under the highest GHG emission scenario. Such a change in climate is likely to modify environmental elements such as temperature and precipitation, which may considerably affect habitat suitability for many species (Allen et al., 2018; Walther et al., 2002; Watson et al., 2012). Even a small change in annual average temperature can have a profound effect upon ecosystem dynamics (Saulnier‐Talbot et al., 2014). The geographical range of the rhinoceros in the past mainly declined due to habitat loss associated with anthropogenic land use changes (Ellis & Talukdar, 2019; Rookmaaker et al., 2016), but our study indicates that future land use change is likely to contribute less to habitat loss than climate change (Appendix S11). Grasslands, which are a vital component of rhinoceros habitat, will substantially decrease globally (Chen et al., 2020). The data on land use change we used in our model also indicate that the extent of farmlands and urban areas will increase and the area of forest and grassland will decrease by the end of this century (Li et al., 2017). The reason behind the comparatively less contribution of land use change in predicted habitat decline is possibly because a majority of alluvial floodplain has already been converted into croplands. Similar studies conducted in India and Nepal for Asian elephant and Himalayan brown bear also suggested that the likely effects of climate change on habitat decline is greater than human land use changes (Dar et al., 2021; Kanagaraj et al., 2019).

The current distribution of rhinoceros based on our ensemble model matched the known occurrence records and is also consistent with the findings of recent research by Jhala et al. (2021). However, a study by Adhikari and Shah (2020) reported that approximately 5% (7240 km^2^) of the country is suitable for rhinoceros, which is greater than our findings. The reason behind this difference is that their model considers a substantial portion of land outside protected areas as suitable rhinoceros habitat, despite these patches being already occupied by human settlements or croplands that will never be converted back to grasslands for rhinoceros conservation. However, their predicted suitable habitat within protected areas seems convincing. For instance, they estimated an area of 659 km^2^ to be suitable for rhinoceros in CNP, similar to our model that estimated 638 km^2^ of suitable habitat within the park. A previous study by Thapa et al. (2014) suggested that 516 km^2^ is currently suitable for rhinoceros in CNP. Ours and each of these studies consistently indicate that suitable rhinoceros habitat is limited to only around 500–700 km^2^ in CNP. Our future ensemble projection also suggests that these parts of CNP are likely to remain prime habitat for rhinoceros in Nepal.

Ecological studies have shown that the rhinoceros population has been gradually shifting to the western parts of CNP in Nepal (Subedi et al., 2013), possibly attributable to a shift in suitable habitat. Our study also indicates a westward expansion of habitat suitability for rhinoceros (Figure 8), given that the extent of predicted loss is more in the central and eastern parts and possible gain in suitable habitat is likely to be more in the western lowlands of Nepal. Our model does show a considerable shift in suitable habitat of rhinoceros within the current distribution range given that 1016 km^2^ of suitable habitat will be lost and 92 km^2^ of new habitat will appear by 2070 under the highest GHG emission scenario. The climate model suggests that annual mean temperature and precipitation are projected to increase in South Asia during the twenty‐first century and the intensity of predicted changes will differ spatially (Almazroui et al., 2020; IPCC, 2014; Jayasankar et al., 2015). One of the possible reasons behind the predicted habitat shift is that the availability and quality of grasslands and wetlands, which are essential components of rhinoceros habitat, are likely to be impacted due to fluctuations in temperature and rainfall. Experimental research on habitat dynamics and fine resolution data on environmental variables in habitat suitability modeling may provide better insights on exact mechanisms of what will make the current suitable habitat unsuitable in future, which is a critical issue for future research.

Our results indicate that the rhinoceros population in Nepal is likely to experience a moderate level of vulnerability to climate change given the predicted loss in suitable habitat under highest GHG emission scenario is 35% by 2070 due to the combined effects of climate and land use changes (Anacker et al., 2013). This result is consistent with the earlier findings of Pant et al. (2020a) on assessing climate change vulnerability to rhinoceros. Thus, our study presents a more optimistic modeling scenario compared to studies on different threatened species in this region. Kanagaraj et al. (2019) predicted that around 42% of currently available habitat for Asian elephants in India and Nepal will be lost due to the combined effects of climate change and human pressure by the end of 2070. Likewise, Dar et al. (2021) suggested that high emission scenarios with land use change may result in a decline of brown bear habitat of >90% by 2070. Mukul et al. (2019) sadly indicated that there will be no suitable habitat for tigers due to the combined effects of sea‐level rise and climate change by 2070 in the Bangladesh Sundarbans.

Despite the habitat constraints faced by rhinoceros in Nepal, the Government of Nepal has proposed the construction of Nijgadh International Airport in an area of 80.50 km^2^ in Kohalbi municipality of Bara district (Shah, 2019)—a place where our model suggests that nearly 33% (26 km^2^) of the area occupied by the proposed airport is currently suitable for rhinoceros. Most of the proposed airport area (94.20%) is forest land including nearly 3 km^2^ of floodplains (Shah, 2019). This area is an important wildlife corridor adjacent to the extended area of PNP, a feeding ground for many mammals and an area frequently utilized by several threatened species including tigers (*Panthera tigris*) and leopards (*Panthera pardus*). Our study also suggests that approximately 27 km^2^ of Rautahat district is suitable habitat for rhinoceros. This area is being used by rhinoceros venturing out from PNP (Acharya & Ram, 2017), and three to four rhinoceroses were recently found in Rautahat district (Rimal et al., 2018). Thus, our model has identified a considerable extent of ecological niche for rhinoceros in Bara and Rautahat districts to the eastern part of PNP, which could serve as additional habitat for rhinoceros conservation. However, threats such as poaching and potential conflict with humans should be addressed while managing this area as an important habitat for rhinoceros and other wildlife species.

In our study, current suitable habitat of 67 km^2^ was detected in KTWR, while the ensemble projection showed that there will be 57 km^2^ of suitable habitat by the end of 2070. The action plan of Nepal Government for rhinoceros conservation (2017–2021) has recommended a feasibility study for translocating rhinoceros in KTWR (DNPWC, 2017). Rhinoceros being a megaherbivore requires large areas of habitat to support viable population (Amin et al., 2006). The average home range size of rhinoceros ranges between 3.5 and 27 km^2^ depending on habitat quality (Dinerstein, 2003; Subedi, 2012). A medium‐sized population of more than 50 is considered a viable population for rhinoceros given that it is less susceptible to extinction and possibly withstand some poaching if supplemented or managed as a metapopulation (Jhala et al., 2021). Considering the habitat suitability as predicted by our ensemble model, KTWR has the potential to support a population of ~45 rhinoceros, but there is no possibility of managing rhinoceros as a metapopulation because the closest suitable habitat as predicted by our model is in Sarlahi district, which is nearly 130 km west from KTWR. It is also important to note that a recent study by Jhala et al. (2021) has suggested that KTWR can hold a minimum of 50 rhinoceros but has not included this protected area as a priority reintroduction site for rhinoceros in Nepal.

We used ensemble SDM to predict the habitat suitability for rhinoceros in Nepal given that it is equally powerful tool as a complex mechanistic model and has been widely used for predicting suitable habitat for species (Fordham et al., 2018). However, SDM is not without limitations. It assumes that species maintain equilibrium with the environment, which may not always be true. Similarly, it does not account for interactions among species which may affect the model accuracy. Thus, these limitations of SDM should be acknowledged while interpreting the findings of this study. In addition, there are uncertainties related to climate and land use change projections. Despite these inherent uncertainties associated with the correlative spatial modeling approach, the present study provides a broad perspective on current ecological niche for rhinoceros in Nepal and where the species is likely to persist in future in the context of likely impacts of climate and land use changes.

## 5 CONCLUSIONS

Our results indicate that rhinoceros in Nepal is likely to face a considerable decrease in habitat suitability over the next 50 years. With an estimated 35% decline in suitable habitat under the highest GHG emission scenario, rhinoceros in Nepal is likely to experience a moderate level of vulnerability due to the combined effects of climate and land use changes, with predicted decline in habitat being influenced to a greater degree by climatic changes than land use changes. Based on the insights provided by our models, literature review, and expert consultation, we have suggested the following conservation measures to moderate the likely impacts arising from climate and land use changes:

- Expand protected areas to secure the predicted climate change refugia for rhinoceros in Nepal. Priority should be given to protect the suitable rhinoceros habitat in Bara, Rautahat, and Sarlahi districts toward the eastern part of Parsa National Park, which could be either managed as an extended area of the existing protected area or declared and managed as a separate protected area.
- Investigate the actual ecological mechanism driving the reduction in currently suitable rhinoceros habitat. Land use changes and the impacts of changing temperature and rainfall on grasslands and wetlands seem particularly obvious, but we were unable to confidently identify other likely mechanisms with our models. We therefore encourage the initiation of experimental on‐ground research and the generation of finer resolution data on environmental variables for further analysis of the habitat suitability to better elucidate these mechanisms and inform rhinoceros conservation interventions.
- Consider the findings of this study while assessing the feasibility of Koshi Tappu Wildlife Reserve as an additional future site for rhinoceros introduction, given that the suitable habitat predicted by our model may not support a viable population of rhinoceros there in long run. In this regard, this research is expected to provide basis for the Department of National Parks and Wildlife Conservation for further assessment and to set priorities for managing the available rhinoceros habitat in the country.
- Avoid suitable rhinoceros habitats when selecting sites for development projects such as airports, railway tracks, and highways given that the current suitable rhinoceros habitat in Nepal is already <2% of the country, and nearly 35% of this current habitat is likely to become unsuitable within a period of 50 years due to the combined effects of climate and land use changes.

## CONFLICT OF INTEREST

None.

## AUTHOR CONTRIBUTIONS

**Ganesh Pant:** Conceptualization (equal); Data curation (lead); Formal analysis (lead); Methodology (equal); Software (lead); Validation (supporting); Visualization (lead); Writing – original draft (lead); Writing – review & editing (equal). **Tek Maraseni:** Conceptualization (equal); Formal analysis (supporting); Methodology (equal); Supervision (lead); Validation (supporting); Visualization (supporting); Writing – review & editing (lead). **Armando Apan:** Conceptualization (equal); Data curation (supporting); Formal analysis (supporting); Methodology (equal); Supervision (supporting); Validation (lead); Writing – review & editing (supporting). **Benjamin L. Allen:** Conceptualization (equal); Formal analysis (supporting); Methodology (equal); Supervision (supporting); Writing – review & editing (supporting).

### OPEN RESEARCH BADGES

This article has earned an Open Data and Open Materials Badges for making publicly available the digitally‐shareable data necessary to reproduce the reported results. The data is available at https://doi.org/10.5061/dryad.wpzgmsbnw.

## Supporting information

Appendix S1‐S11

## Data Availability

Dataset and R Markdown File related to ensemble modeling used in this study are deposited in Dryad Digital Repository and are available via the following link. https://doi.org/10.5061/dryad.wpzgmsbnw.
