# Supplementary material for: Predicted declines in suitable habitat for greater one‐horned rhinoceros (Rhinoceros unicornis) under future climate and land use change scenarios
Source: Ecol Evol. 2021 Dec 7;11(24):18288–304. doi: 10.1002/ece3.8421 (PMC8717310; doi:10.1002/ece3.8421)
Supplement: Supplementary file 1 — Appendix S1‐S11 [file ECE3-11-18288-s001.docx]

Appendices for

**Predicted declines in suitable habitat for greater one-horned rhinoceros *(Rhinoceros unicornis)* under future climate and land use change scenarios**

**Appendix S1.** List of environmental variables that are possibly determining the habitat selection of greater one-horned rhinoceros (*Rhinoceros unicornis*).

| Category | Source | Selected variables | | | Resolution | Type |
| --- | --- | --- | --- | --- | --- | --- |
| Bioclimatic | WORLDCLIM | BIO1– Annual mean temperature | | | ~1 km | Continuous |
|  |  | BIO2– Mean Diurnal Range (Mean of monthly (Maximum temperature – minimum temperature)) | | | ~1 km | Continuous |
|  |  | BIO3– Isothermality (BIO2/BIO7) (×100) | | | ~1 km | Continuous |
|  |  | BIO4– Temperature seasonality (Standard deviation x 100) | | | ~1 km | Continuous |
|  |  | BIO5– Maximum temperature of warmest month | | | ~1 km | Continuous |
|  |  | BIO6– Minimum temperature of coldest month | | | ~1 km | Continuous |
|  |  | BIO7– Temperature annual range (BIO5 – BIO6) | | | ~1 km | Continuous |
|  |  | BIO8–Mean temperature of wettest quarter | | | ~1 km | Continuous |
|  |  | BIO9 - Mean temperature of driest quarter | | | ~ 1 km | Continuous |
|  |  | BIO10– Mean temperature of warmest quarter | | | ~1 km | Continuous |
|  |  | BIO11– Mean temperature of coldest quarter | | | ~1 km | Continuous |
|  |  | BIO12 - Annual precipitation | | | ~1 km | Continuous |
|  |  | BIO13– Precipitation of wettest month | | | ~1 km | Continuous |
|  |  | BIO14 - Precipitation of the driest month | | | ~1 km | Continuous |
|  |  | BIO15– Precipitation seasonality (Coefficient of variation) | | | ~1 km | Continuous |
|  |  | BIO16– Precipitation of wettest quarter | | | ~1 km | Continuous |
|  |  | BIO17– Precipitation of driest quarter | | | ~1 km | Continuous |
|  |  | BIO18– Precipitation of warmest quarter | | | ~1 km | Continuous |
|  |  | BIO19– Precipitation of coldest quarter | | | ~1 km | Continuous |
| Topographic and habitat | SRTM | Elevation | | | ~ 30 m | Continuous |
|  |  | Aspect | | | ~ 30 m | Continuous |
|  |  | Slope | | | ~ 30 m | Continuous |
|  | ESRI 2020 Land Cover | Distance from grasslands | | | ~1o m | Continuous |
|  |  | Distance from wetlands | | | ~10 m | Continuous |
|  |  | Distance from forests | | | ~10 m | Continuous |
| Anthropogenic | MODIS Land Cover | Croplands | | | ~500 m | Continuous |
|  | HDX | Population density | | | ~1 km | Continuous |
|  | GEOFABRIK | Distance from roads | | | ~1 km | Continuous |
|  |  | |  |  | |  |

**Appendix S2.** List of environmental variables retained after multicollinearity test.

| Category | Source | Selected variables | | | Resolution | Type |
| --- | --- | --- | --- | --- | --- | --- |
| Bioclimatic | WORLDCLIM | BIO2– Mean Diurnal Range (Mean of monthly (Maximum temperature – minimum temperature)) | | | ~1 km | Continuous |
|  |  | BIO3– Isothermality (BIO2/BIO7) (×100) | | | ~1 km | Continuous |
|  |  | BIO9 - Mean temperature of driest quarter | | | ~ 1 km | Continuous |
|  |  | BIO12 - Annual precipitation | | | ~1 km | Continuous |
|  |  | BIO14 - Precipitation of the driest month | | | ~1 km | Continuous |
|  |  | BIO15– Precipitation seasonality (Coefficient of variation) | | | ~1 km | Continuous |
|  |  | BIO18– Precipitation of warmest quarter | | | ~1 km | Continuous |
|  |  | BIO19– Precipitation of coldest quarter | | | ~1 km | Continuous |
| Topographic and habitat | SRTM | Slope | | | ~ 30 m | Continuous |
|  | Esri 2020 Land Cover | Distance from grasslands | | | ~10 m | Continuous |
|  |  | Distance from wetlands | | | ~10 m | Continuous |
|  |  | Distance from forests | | | ~10 m | Continuous |
| Anthropogenic | MODIS Land Cover | Croplands | | | ~ 500 m | Continuous |
|  | HDX | Population density | | | ~1 km | Continuous |
|  |  | |  |  | |  |

**Appendix S3.** Checklist for ODMAP (Overview, data, model, assessment, and prediction) protocol while developing habitat suitability models for greater one-horned rhinoceros (*Rhinoceros unicornis*) in Nepal.

| **ODMAP element** | **Contents** |
| --- | --- |
| **OVERVIEW** | |
| *Authorship* | - **Authors:** Ganesh Pant, Tek Maraseni, Armando Apan and Benjamin L. Allen - **Contact e-mail:** [ganeshpant@yahoo.com](mailto:ganeshpant@yahoo.com), [ganesh.pant@usq.edu.au](mailto:ganesh.pant@usq.edu.au) - **Title:** Predicted declines in suitable habitat for greater one-horned rhinoceros (*Rhinoceros unicornis*) under future climate and land use change scenarios |
| *Model objective* | - **Objective:** Predict habitat suitability - **Target outputs:** Current and future habitat suitability maps |
| *Taxon* | Greater one-horned rhinoceros, *Rhinoceros unicornis*, Rhinocerotidae, Perissodactyla, Mammalia |
| *Location* | Nepal, Asia |
| *Scale of analysis* | - **Spatial extent (Lon/Lat):** 80^0^04’ - 88^0^12’ E, 26^0^22’ - 30^0^27’ N, covering 1,47,516 km^2^ - **Spatial Resolution:** 1 km - **Temporal extent/time period:** Species occurrence data- 2008 to present; environmental data - 1970 to present, and future projection (2050 and 2070) - **Type of extent boundary:** Political (the Federal Democratic Republic of Nepal) |
| *Biodiversity data overview* | - **Observation type:** Standard monitoring, field survey, GPS tracking - **Response/Data type:** Presence-only |
| *Type of predictors* | Bioclimatic, anthropogenic, topographic and habitat variables |
| *Conceptual model / hypothesis* | - **Hypothesis about species-environment relationships:** Species maintain equilibrium with their environment. The distribution of rhinoceros is determined by climatic factors (temperature and precipitation) and the presence of a specific habitat component (grasslands and wetlands), constrained by topographic factors (elevation, aspect, and slope), and influenced by anthropogenic disturbances (land use land cover and population density). |
| *Assumptions* | - Species are at equilibrium with their environment and do not occur elsewhere. - Species occurrence data are free from observational bias and any biases are accounted for or corrected. - Key predictor variables of the species are available and incorporated in the model. - Predictor variables are measured or estimated without error |
| *SDM algorithms* | - **Algorithms:** We used ten SDM algorithms available in BIOMOD2 as follows  1. Artificial Neural Network (ANN) 2. Classification Tree Analysis (CTA) 3. Flexible Discriminant Analysis (FDA) 4. Generalised Additive Model (GAM) 5. Generalised Boosting Model (GBM) 6. Generalised Linear Model (GLM) 7. Multiple Adaptive Regression Splines (MARS) 8. Maximum Entropy (MAXENT) 9. Random Forest (RF) 10. Surface Range Envelope (SRE)  - **Model complexity:** We chose ten different modelling algorithms to yield complex response surfaces but prevent overfitting. - **Model averaging:** We selected all models from ten SDM algorithms having a True Skill Statistics (TSS) value >0.85 for building ensemble model using the weighted mean approach. |
| *Model workflow* | - We compiled rhinoceros presence data from all possible sources. - We used spThin package in R (Aiello‐Lammens et al., 2015) to spatially rarefy presence data to reduce sample bias (Boria et al., 2014) and used a dataset of 495 selected rhinoceros presence points. - We identified a set of 28 environmental variables primarily based on literature suggesting the significance of these variables for rhinoceros habitat suitability (Laurie, 1982; Dinerstein & Price, 1991; Jnawali, 1995; Dinerstein, 2003; Pradhan et al., 2008; Subedi, 2012; Pant et al., 2020). - We then excluded the variables with correlation coefficients >0.8 and variance inflation factor (VIF) >5 after testing the multicollinearity among environmental variables using the USDM (Uncertainty Analysis for Species Distribution Models) package in R to avoid model overfitting (Gareth et al., 2013; Naimi et al., 2014), retaining 14 variables for further analysis. - We selected nine of these as ecologically meaningful variables following a reiterative process of model formation and stepwise removal of the least contributing variables, as suggested by Zeng et al. (2016). - We generated pseudo-absence data (n=10,000) and repeated pseudo-absence generation three times to avoid random bias (Barbet-Massin et al., 2012). - We divided rhinoceros presence and pseudo-absence data into training (80%) and testing data (20%). - After preparing appropriate data layers, we ran a total of 90 models comprising ten SDM algorithms, three pseudo-absence selection and three evaluation runs. - We generated ensemble model using the ensemble modelling function in BIOMOD2. We included all the models having TSS value >0.85 for building ensemble model. - We projected the ensemble models for two different climate scenarios for 2050 and 2070. - Finally, we employed range size function within the BIOMOD2 package for calculating the range shifts. - The model workflow is also depicted in Figure **3** in the paper. |
| *Software, codes, and data* | - **Modelling platform:** R (Version 4.1.1) with package BIOMOD2 - **Code:** Code is shared in specified data repository - **Data:** Data is shared in specified data repository |
| **DATA** | |
| *Biodiversity data* | - **Taxon names:** *Rhinoceros unicornis* - **Taxonomic reference system:** N/A - **Ecological level:** Species level - **Data source:**  1. Rhinoceros presence records from Government Department: Compiled from rhinoceros census and monitoring of individual rhinoceros using GPS collar between 2008 and 2017. 2. Field work for this research: Rhinoceros presence points recorded in April 2019 using handheld GPS unit. 3. GBIF website: Downloaded in February 202o.  - **Sampling design:** N/A - **Sample size:** We compiled the rhinoceros presence data from entire Nepal. - **Absence data:** We used presence-only data for modelling given that we did not have true absence data for rhinoceros. Pseudo-absence data were generated for running models in BIOMOD2. - **Data cleaning and filtering:** We used the SpThin package in R to spatially rarefy the occurrence dataset (Aiello‐Lammens et al., 2015). Spatial filtering reduces the effects of sample bias and helps to improve the predictive performance of the models (Boria et al., 2014). |
| *Data partitioning* | - Rhinoceros presence and pseudo-absence data were split into training (80%) and testing data sets (20%). |
| *Predictor variables* | - **Predictor variables:**  1. Bioclimatic variables — Temperature annual range (BIO7), mean temperature of driest quarter (BIO9), and annual precipitation (BIO12) 2. Topographic variables — Slope 3. Habitat variables — Distance from grasslands, distance from wetlands, and distance from forests 4. Anthropogenic variables — Croplands and population density  - **Data source:**  1. <https://www.worldclim.org/> 2. <https://www.usgs.gov/science-explorer-results?es=SRTM> 3. <https://www.arcgis.com/home/item.html?id=d6642f8a4f6d4685a24ae2dc0c73d4ac> 4. <https://doi.org/10.5067/MODIS/MCD12Q1.006>, <http://www.geosimulation.cn/flus.html>,   https://data.humdata.org/dataset   - **Data processing:** - We downloaded the data layers from free online sources and standardised these data using various functions in ArcMap 10.8.1 (ESRI, 2017). - We extracted the grass, water and trees layers of the study area from Esri 2020 Land Cover Raster Dataset in ArcMap 10.8.1 (ESRI, 2017). We converted the raster data into polygon and generated data layers containing proximity to grasslands, wetlands and forests using Euclidean Distance tool in ArcMap 10.8.1 (ESRI, 2017). - We resampled raster data of the environmental variables in ArcMap 10.8.1 (ESRI, 2017) at a spatial resolution of 1 km. We used bilinear interpolation method as environmental variables we used were continuous data. - **Spatial resolution of raw data:** 10m, 30 m, 300 m, 500 m, 1 km - **Projection:** WGS84 |
| **MODEL** | |
| *Variable pre-selection* | - We identified a set of 28 environmental variables primarily based on literature suggesting the significance of these variables for rhinoceros habitat suitability (Laurie, 1982; Dinerstein & Price, 1991; Jnawali, 1995; Dinerstein, 2003; Pradhan et al., 2008; Subedi, 2012; Pant et al., 2020). - After multicollinearity test among environmental variables, we excluded the variables with correlation coefficients >0.8 and variance inflation factor (VIF) >5 and retained 14 variables for further analysis. - We selected nine of these as ecologically meaningful variables following a reiterative process of model formation and stepwise removal of the least contributing variables, as suggested by Zeng et al. (2016). |
| *Multicollinearity* | - We tested multicollinearity among environmental variables using the USDM (Uncertainty Analysis for Species Distribution Models) package in R to avoid model overfitting (Gareth et al., 2013; Naimi et al., 2014) |
| *Model settings* | - We used default settings for BIOMOD2 to run models using ten SDM algorithms. |
| *Model estimates* | - **Model coefficient:** We used TSS to evaluate the predictive performance while we analysed ROC for cross-comparison. In addition, we used Boyce index for cross validation. - **Variable importance:** We calculated the variable importance of the ensemble model and found that five environmental variables — distance from grasslands, annual precipitation, mean temperature of driest quarter, distance from wetlands, and slope, contributed the most in the model. |
| *Model averaging / ensembles* | - We selected all models from ten SDM algorithms having a True Skill Statistics (TSS) value >0.85 for building ensemble model using the weighted mean approach. |
| *Non-independence* | - We did not perform any test for checking non-independence of the models. |
| *Threshold selection* | - Binary predictions were derived by using the TSS maximisation threshold. |
| **ASSESSMENT** | |
| *Performance statistics* | - **Performance statistics estimated on training data:** We assessed model performance based on TSS value from 90 model runs. |
| *Plausibility checks* | - **Response plots:** We generated the response curves of the best performing model and analysed it for ecological plausibility. For instance, areas with >1500 mm of average annual rainfall was suitable for rhinoceros as indicated by the response curve. |
| **PREDICTION** | |
| *Prediction output* | - We used continuous predictions of occurrence probability for rhinoceros further analysis of habitat suitability as well as predicted presence generating the binary map (presence-absence map) using the optimal prediction value threshold of 0.376 identified automatically based on TSS value. |
| *Uncertainty quantification* | - **Algorithmic uncertainty:** Ensemble forecasting can reduce model-based uncertainty in prediction from SDMs (Araújo & New, 2007). Thus, we accounted for algorithmic uncertainty by developing an ensemble model from all ten SDM algorithms based on consensus method for combining output of single models (Marmion et al., 2009). - **Reality check:** We further endeavoured to validate the on-ground reality of the current habitat suitability model for rhinoceros in Nepal through expert consultation. For this, we shared the current habitat suitability model we generated to five field biologists each having more than ten years of professional experience in research and management of rhinoceros in Nepal. All of them agreed that the current suitability model has captured both currently occupied and other possible habitat of rhinoceros in Nepal. |

**Appendix S4.** Predictive performance of different algorithms using testing data for modelling habitat suitability of greater one-horned rhinoceros (*Rhinoceros unicornis*) in Nepal.


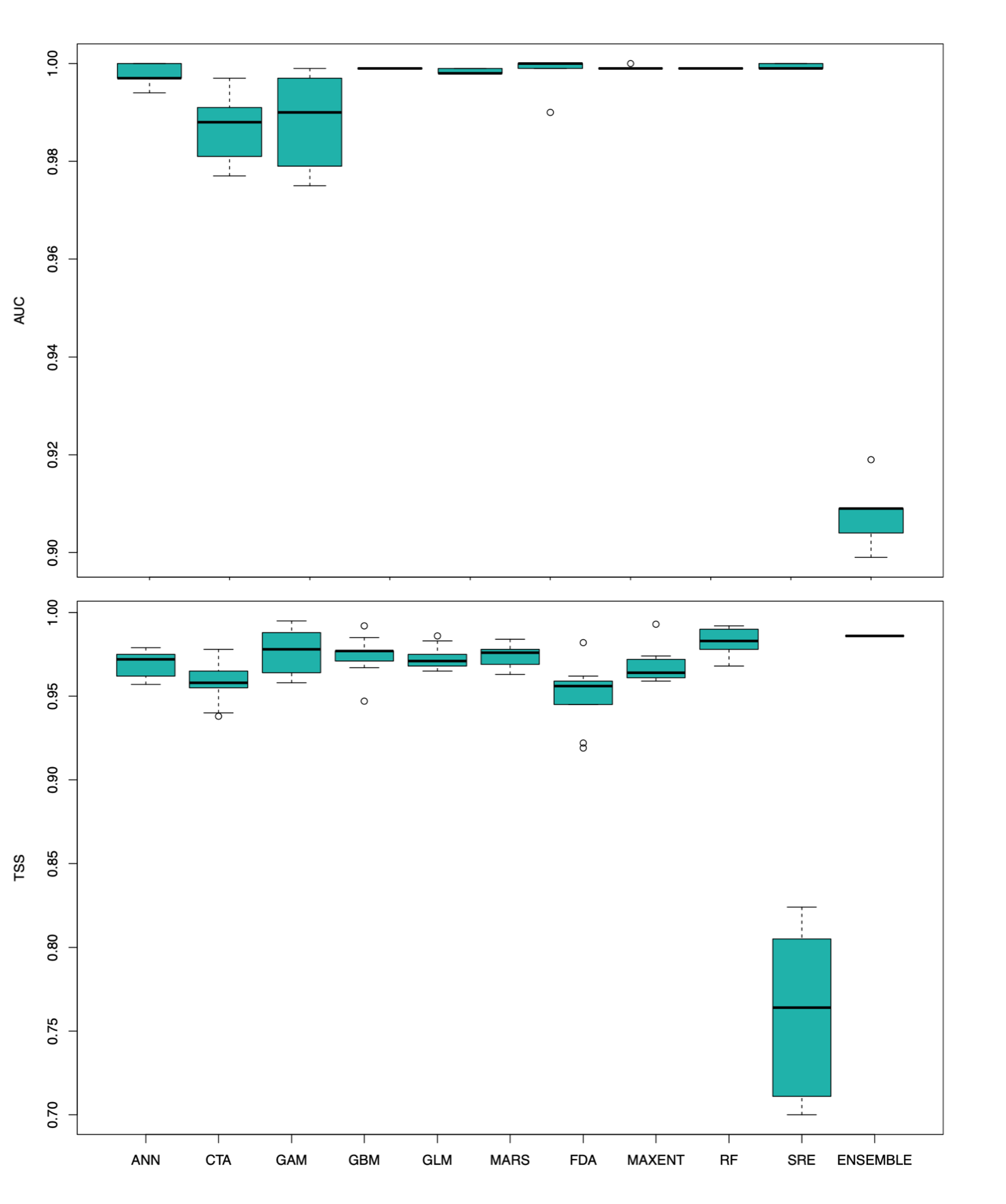


**Appendix S5.** Predictive performance of different algorithms using evaluating data for modelling habitat suitability of greater one-horned rhinoceros (*Rhinoceros unicornis*) in Nepal.

**Appendix S6.** Predictive performance of the models included in the ensemble model based on Area Under Curve (AUC) value and the Boyce index for modelling habitat suitability of greater one-horned rhinoceros (*Rhinoceros unicornis*) in Nepal.


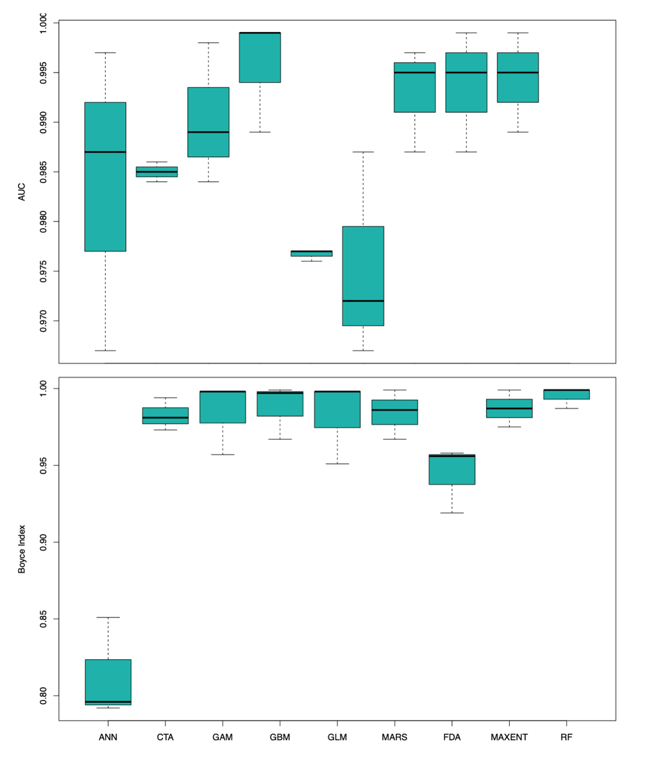


**Appendix S7.** Distribution of current suitable habitat for greater one-horned rhinoceros (*Rhinoceros unicornis*) in and outside protected areas (PAs) of Nepal.


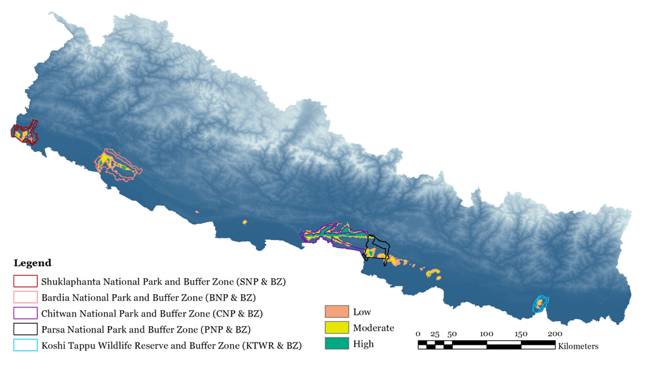


**Appendix S8.** Estimated area of current suitable habitat for greater one-horned rhinoceros (*Rhinoceros unicornis*) in and outside protected areas (PAs) of Nepal.


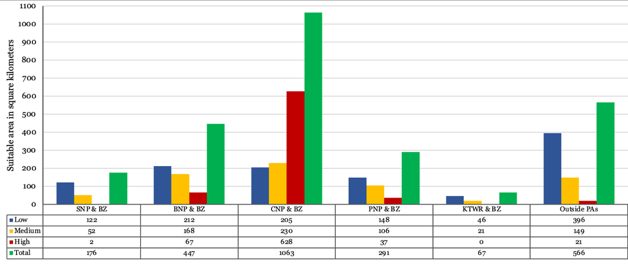


**Appendix S9.** Distribution of current suitable habitat for greater one-horned rhinoceros (*Rhinoceros unicornis*) in different districts of Nepal.


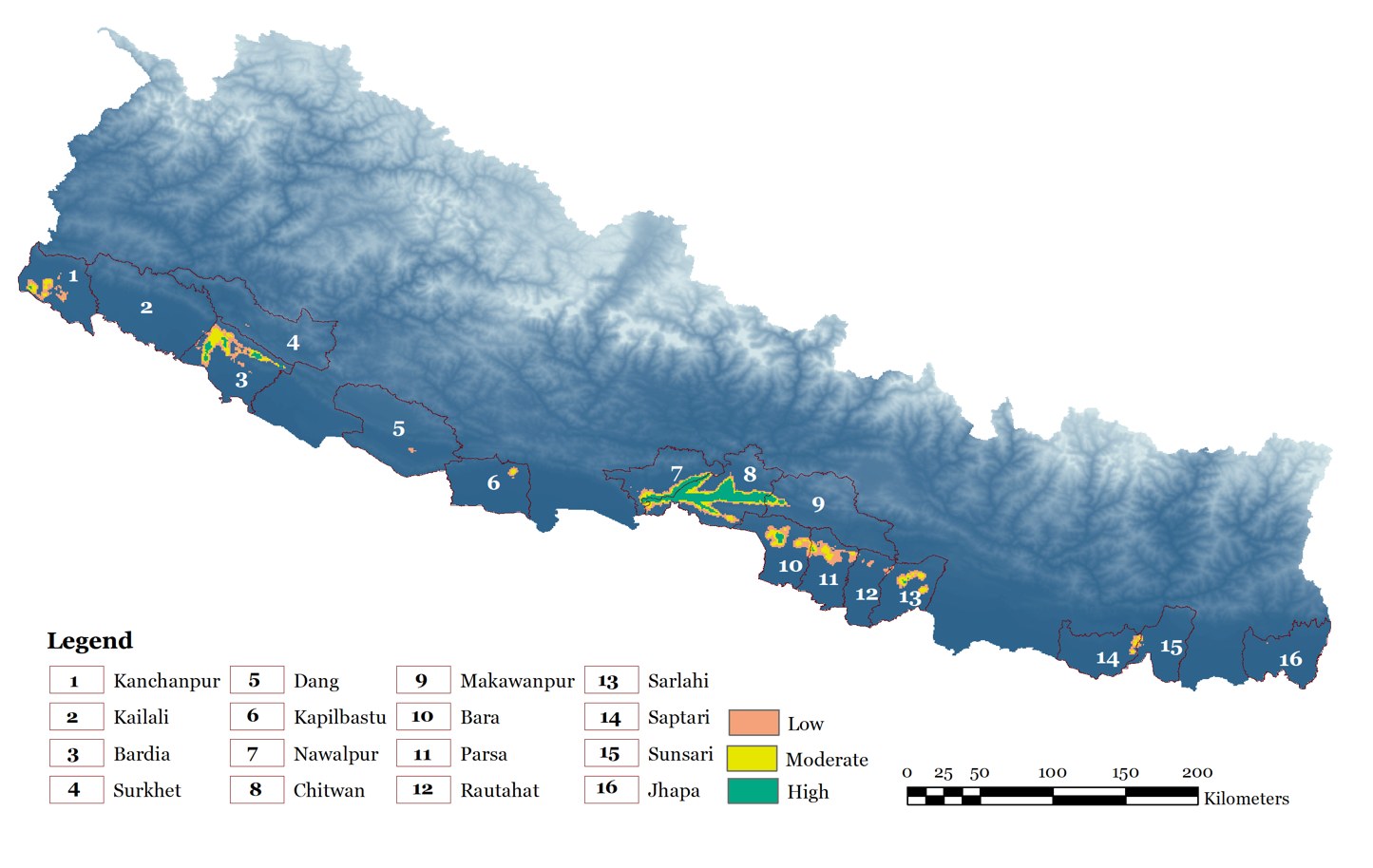


**Appendix S10.** Estimated area of current suitable habitat for greater one-horned rhinoceros (*Rhinoceros unicornis*) in different districts of Nepal.


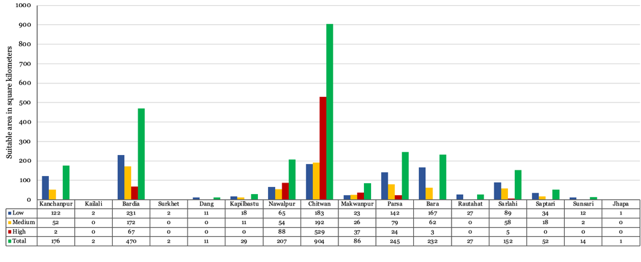


**Appendix S11.** Percentage change in habitat suitability for greater one-horned rhinoceros in Nepal predicted by the ensemble model in different climate and land use change scenarios. a. Climate change only and b. Land use change only. SSP1-2.6 and SSP5-8.5 are two different climate change scenarios that anticipate a mean warming of 2^0^C and 5.5^0^C by 2100, respectively. A1B scenario – Moderate increase in land use across all resources and A2 scenario – High emphasis on development with adverse impact on the environment.

| a.  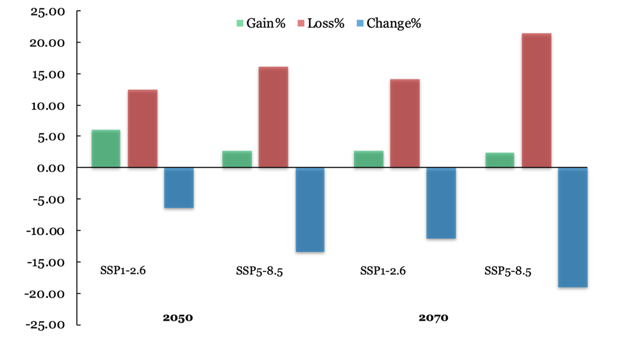 |
| --- |
| b.  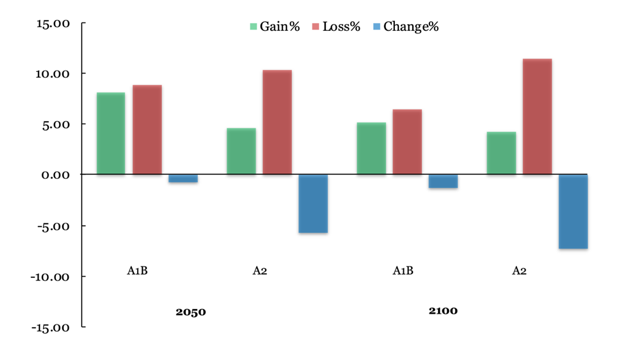 |
